# Wnt-related SynGAP1 is a neuroprotective factor of glutamatergic synapses against Aβ oligomers

**DOI:** 10.3389/fncel.2015.00227

**Published:** 2015-06-15

**Authors:** Juan F. Codocedo, Carla Montecinos-Oliva, Nibaldo C. Inestrosa

**Affiliations:** ^1^Molecular Neurobiology Lab and Center for Aging and Regeneration Center, Department of Cell and Molecular Biology, Faculty of Biological Sciences, Pontifical Catholic University of ChileSantiago, Chile; ^2^Center for Healthy Brain Ageing, School of Psychiatry, Faculty of Medicine, University of New South WalesSydney, NSW, Australia; ^3^Centro de Excelencia en Biomedicina de Magallanes, Universidad de MagallanesPunta Arenas, Chile; ^4^Centro UC Síndrome de Down, Pontificia Universidad Católica de ChileSantiago, Chile

**Keywords:** Wnt-5a, microRNAs, SynGAP, CamKII, Alzheimer disease

## Abstract

Wnt-5a is a synaptogenic factor that modulates glutamatergic synapses and generates neuroprotection against Aβ oligomers. It is known that Wnt-5a plays a key role in the adult nervous system and synaptic plasticity. Emerging evidence indicates that miRNAs are actively involved in the regulation of synaptic plasticity. Recently, we showed that Wnt-5a is able to control the expression of several miRNAs including miR-101b, which has been extensively studied in carcinogenesis. However, its role in brain is just beginning to be explored. That is why we aim to study the relationship between Wnt-5a and miRNAs in glutamatergic synapses. We performed *in silico* analysis which predicted that miR-101b may inhibit the expression of synaptic GTPase-Activating Protein (SynGAP1), a Ras GTPase-activating protein critical for the development of cognition and proper synaptic function. Through overexpression of miR-101b, we showed that miR-101b is able to regulate the expression of SynGAP1 in an hippocampal cell line. Moreover and consistent with a decrease of miR-101b, Wnt-5a enhances SynGAP expression in cultured hippocampal neurons. Additionally, Wnt-5a increases the activity of SynGAP in a time-dependent manner, with a similar kinetic to CaMKII phosphorylation. This also, correlates with a modulation in the SynGAP clusters density. On the other hand, Aβ oligomers permanently decrease the number of SynGAP clusters. Interestingly, when neurons are co-incubated with Wnt-5a and Aβ oligomers, we do not observe the detrimental effect of Aβ oligomers, indicating that, Wnt-5a protects neurons from the synaptic failure triggered by Aβ oligomers. Overall, our findings suggest that SynGAP1 is part of the signaling pathways induced by Wnt-5a. Therefore, possibility exists that SynGAP is involved in the synaptic protection against Aβ oligomers.

## Introduction

Dendritic spines compartmentalize biochemical cascades at the postsynaptic level and are enriched in neurotransmitter receptors, ion channels, and components of various signaling pathways (Chen and Sabatini, 2012; Colgan and Yasuda, 2014), including the Wnt signaling pathway (Inestrosa and Arenas, 2010; Budnik and Salinas, 2011).

The signaling pathways mediated through Wnt have been implicated in various cellular processes, including cell proliferation, migration, establishment of polarity, cell fate specification, and adult hippocampal neurogenesis (Lie et al., 2005; Nusse and Varmus, 2012; Varela-Nallar and Inestrosa, 2013). Therefore, deregulation of Wnt signaling has been associated with the development of several diseases, including autism (Zhang et al., 2012; Sowers et al., 2013), schizophrenia (Lovestone et al., 2007; Inestrosa et al., 2012), and Alzheimer disease (AD; Inestrosa and Toledo, 2008; Purro et al., 2012; Inestrosa and Varela-Nallar, 2014). Recently, a number of studies have highlighted a role for Wnt signaling in synaptic formation and function (Gogolla et al., 2009; Ciani et al., 2011; Muñoz et al., 2014). In particular, we showed that the Wnt-5a ligand exerts important effects in the postsynaptic region of hippocampal neurons. For example, Wnt-5a stimulation increases the: GABAa receptor recycling (Cuitino et al., 2010), the clustering of postsynaptic density (PSD) protein 95 (PSD-95; Farías et al., 2009) and the density of dendritic spines (Varela-Nallar et al., 2010). Moreover, in hippocampal slices, Wnt-5a modulates synaptic activity through the enhancement of long-term potentiation (LTP; Cerpa et al., 2011; Vargas et al., 2014). These findings indicate that Wnt-5a regulates the assembly and function of the excitatory postsynaptic region of central synapses (Inestrosa and Varela-Nallar, 2014). However, the mechanism underlying these effects is still elusive.

Recently, we explore a new mechanism of regulation mediated by Wnt-5a that includes the modulation of microRNAs (miRNAs). MiRNAs are a family of small non-coding RNAs, that control the gene expression of their targets through base pairing between the 3′ untranslated region (UTR) of mRNA and miRNA “*seed”* region at the 5′ end, thereby inhibiting the translation of the target proteins (Bartel, 2009). We identified more than 30 miRNAs with differential expression after 1 h treatment with Wnt-5a in cultured rat hippocampal neurons and miR-101b was the most affected miRNA after Wnt-5a signaling activation (Codocedo and Inestrosa, 2013). While the role of miR-101b has been thoroughly investigated in cancerogenesis (Strillacci et al., 2009; Hao et al., 2011; He et al., 2012), the role for this miRNAs in the brain has just begun to emerge. It was reported that miR-101b regulates the expression of the amyloid precursor protein (APP; Vilardo et al., 2010; Long and Lahiri, 2011; Barbato et al., 2014), ataxin1 (Lee et al., 2008), and the Fragile X Mental Retardation gene 1 (FMR1; Zongaro et al., 2013) in the hippocampus.

Among the many targets predicted for miR-101b, we focused on SynGAP (Synaptic GTPase-Activating Protein), because it is an abundant key PSD signaling enzyme. It negatively regulates small G protein signaling downstream of glutamate receptor activation and is related to the regulation of synapse density, dendritic spine shape, and synaptic physiology (McMahon et al., 2012). Conversely, alterations in SynGAP functions has been linked to intellectual disability (ID) and autism spectrum disorders (ASDs) (Berryer et al., 2013). Additionally, several reports suggest that the upstream activator of SynGAP is Ca^2+^/calmodulin-dependent protein kinase II (CaMKII) which in turn is activated by increased calcium levels mediated by *N*-methyl-D-aspartic acid receptors (NMDAR) activation (Krapivinsky et al., 2004; Rumbaugh et al., 2006; Araki et al., 2015). Since Wnt-5a is able to down-regulate a miRNA that targets SynGAP *in silico* and activates the Wnt/Ca^2+^ signaling pathway in the dendritic compartments of mature hippocampal neurons (Varela-Nallar et al., 2010), we evaluate whether SynGAP is part of the mechanism by which Wnt-5a induces changes at the post-synaptic region. Considering the neuroprotective effects of this ligand against amyloid-β oligomers (Aβ oligomers; Cerpa et al., 2010; Varela-Nallar et al., 2012), we explore the effects of Wnt-5a over SynGAP, in the presence of Aβ oligomers.

## Materials and Methods

### Ethics Statement

Sprague–Dawley rats were housed in the University Animal Facility and handled according to the guidelines outlined and approved through the Institutional Animal Care and Use Committee at the Faculty of Biological Sciences of the Pontificia Universidad Católica de Chile, and following the guidelines of the American Physiological Society Rockville, MD.

### Primary Culture of Rat Hippocampal Neurons

Rat hippocampal cultures were prepared as previously described (Alvarez et al., 2004; Kaech and Banker, 2006). Primary hippocampal neurons were obtained from 18-days-old Sprague–Dawley rat embryos and maintained in Dulbecco’s modified Eagle’s medium (DMEM) supplemented with 10% horse serum for 2 h. The culture medium was subsequently substituted with Neurobasal medium supplemented with B27, 100 μg/ml streptomycin, and 100 units/ml penicillin. At 3 days *in vitro* (DIV), the cells were treated with 2 μM araC for 24 h to reduce the number of glial cells present in the culture. For western blot (WB) analyses, 400,000 cells per well were seeded, and for immunofluorescence studies, 35,000 cells were plated per well. At 14 DIV, the neurons were stimulated with 300 ng/mL of recombinant Wnt-5a (rWnt-5a; R&D System, Minneapolis, MN, USA) resuspended in Neurobasal medium. Incubations were conducted at 37°C.

### HT22 Cell Line

HT22 murine hippocampal neuronal cells were maintained in DMEM supplemented with 10% fetal bovine serum, 100 μg/ml streptomycin, and 100 units/ml penicillin, high glucose and incubated at 37°C under 5% CO_2_ as previously described (Chhunchha et al., 2013). Transfections were performed after 2 days at approximately 60% confluency.

### Bioinformatics

For computational prediction of the miRNA targets, we used the TargetScan web platform (Lewis et al., 2005), which predicts biological targets of miRNAs by searching for the presence of conserved 8- and 7-mer sites that match the seed region of each miRNA. In addition, TargetScan examines the binding sites for thermodynamic stability using RNAfold from the Vienna RNA Package (Hamzeiy et al., 2014).

### miR-101b Gain-of-Function

For miR-101b gain-of-function, we used mirVana miRNA mimic (Life Technologies, Carlsbad, CA, USA), which represents a partially double-stranded RNA that mimics endogenous precursor miRNA and is processed to form an active miRNA molecule that targets specific mRNAs (Nicoloso et al., 2009). For miR-101b gain-of-function in HT-22 cells, Lipofectamine 2000 reagent (Invitrogen, Karlsruhe, Germany) was used according to the manufacturer’s protocol. At 48 h post-transfection, the cells were used for WB analysis. For controls conditions HT-22 cells were transfected with mirVana miRNA Mimic Negative Control #1 (Life Technologies, Carlsbad) which correspond to a random sequence miRNA mimic molecule that not produce identifiable effects on known miRNA function.

### Western Blot Analysis

The extraction of total protein from cell culture of hippocampal neurons and immunoblot analysis were performed as previously described (Varela-Nallar et al., 2009; Codocedo et al., 2012). The following primary antibodies were used: rabbit anti-pSynGAP (1:1.000; ABCAM), rabbit anti-SynGAP (1:1.000; ABCAM), mouse anti pCAMKII (1:1.000, Santa Cruz), and anti GAPDH (1:10.000, Santa Cruz). Primary antibodies were recognized using either a horseradish peroxidase (HRP)-conjugated goat anti-rabbit antibody (1:7000, Thermo Scientific) or an HRP-conjugated rabbit anti-mouse antibody (1:7.000 Thermo Scientific). The secondary antibodies were detected through enhanced chemiluminescence using the ECL Plus Western blotting detection system (GE Healthcare). Densitometric analysis was performed using NIH ImageJ software.

### Immunofluorescence and Microscopy

Immunofluorescence studies were performed as previously described (Varela-Nallar et al., 2009; Codocedo et al., 2012). Briefly, 14–16 DIV hippocampal neurons were depleted with Neurobasal medium (Invitrogen Corporation, Carlsbad, CA, USA) 1 h before treatment. Then, cells were treated *in vitro* with 300 ng/mL rWnt-5a and/or 1 μM Aβ1-42 oligomers following a time curve (0, 15, 30, 60, and 120 min). In every case, cells were kept at 37°C in incubator with 95% O_2_. Following treatment, cells were washed three times with PBS Ca^2+/^Mg^2+^ and fixated with paraformaldehyde 4%–sucrose 4%. After another round of washes, cells were permeabilized with PBS Ca^2+/^Mg^2+^ + Triton X 0.2%. Then, cells were immunolabeled with anti-SynGAP antibody (1:500, ABCAM) overnight at 4°C. On the next day, cells where washed as previously stated, and incubated with Alexa-conjugated secondary antibodies for 30 min at 37°C and Höescht staining (1:1000) for 10 min at room temperature. Coverslips were mounted using Fluoromont G mounting media and analyzed on an Olympus Takyo Japan Fluoview FV 1000 confocal microscope.

### Aβ_1-42_ Oligomers Preparation

A lyophilized stock peptide was resuspended in anhydrous sterile dimethyl sulfoxide (DMSO), to form 5 mM aliquots that were immediately frozen. Aliquotes were diluted in PBS, pH 7.4 to a final concentration of 100 μM and stirred continuously approximately at 1350 rpm for 1 h at room temperature. Final concentrations for immunofluorescence studies were 5 μM Aβ oligomers and 0.02% DMSO. Control of the monomers to tetramers oligomeric (low molecular weight) species present after following this protocol can be found in previous publications from our group (Silva-Alvarez et al., 2013).

### Statistical Analysis

All data were analyzed statistically with Prism 5 (Prism GraphPad Software, GraphPad Software Inc., La Jolla, CA, USA) using one-way ANOVA, followed by Dunn’s Multiple Comparison Test. The error bars indicate SEM. A *p* < 0.05 was considered statistically significant.

## Results

### SynGAP is a Target of miR-101b, a Wnt-5a-Regulated microRNA

**FIGURE 1:**
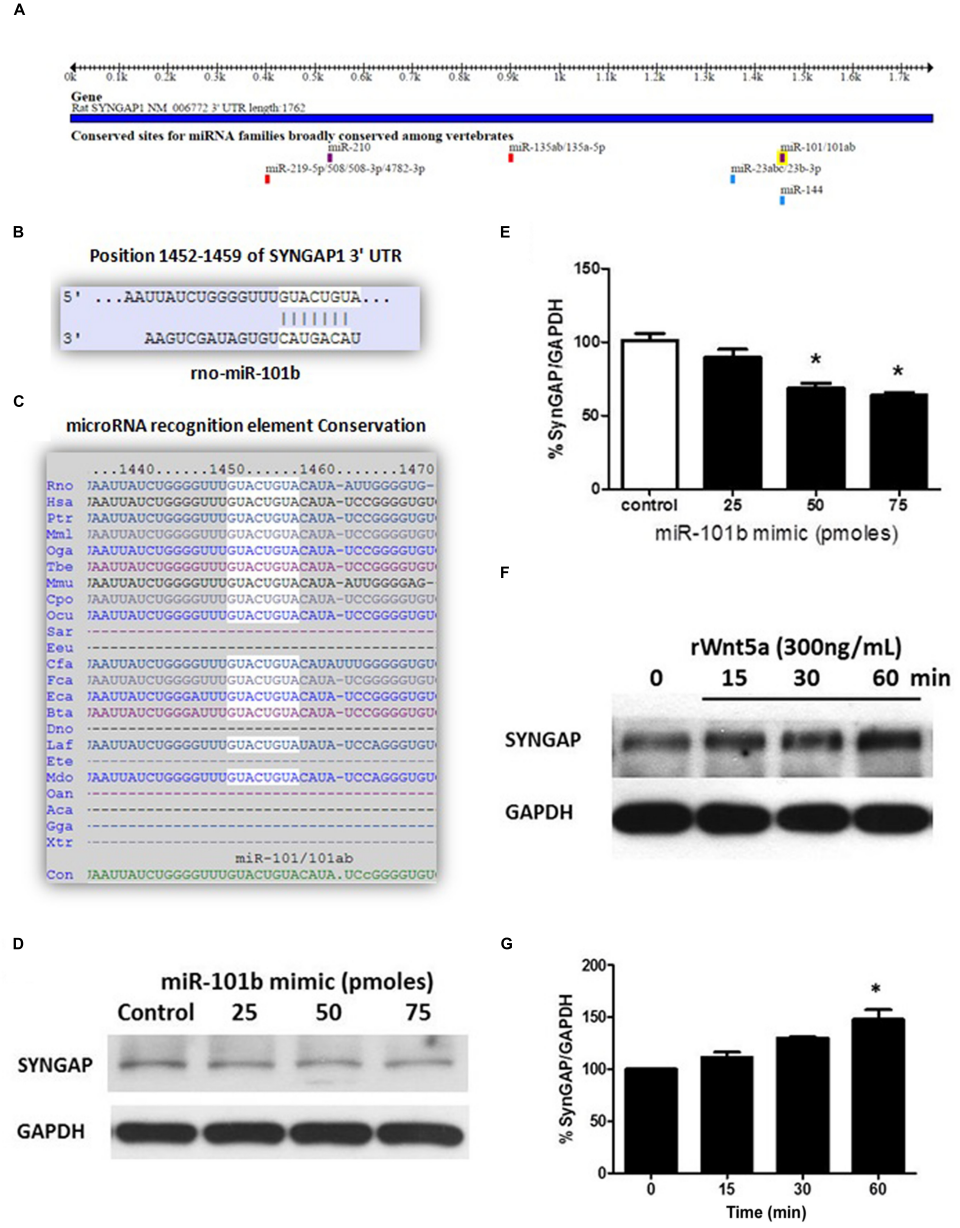
**Synaptic GTPase-Activating Protein (SynGAP) is a target of miR-101b, a Wnt-5a-regulated microRNA. (A)** Predicted binding site for the seed sequence of miR-101b in the 3′UTR of SynGAP. Obtained from TargetScan. **(B)** Complementarity of the sequences between miR-101b and the SynGAP 3′-UTR of rat genome obtained from TargetScan. **(C)** Degree of conservation in mammalian species obtained from TargetScan. **(D)** Endogenous levels of SynGAP are significantly decreased through gain-of function of miR-101b mimics in HT22 cells. **(E)** Densitometric analysis of the western blot (WB) shown in **(D)**. **(F)** WB analysis of total SynGAP levels in hippocampal neurons treated with recombinant Wnt-5a (300 ng/mL) at different time points. **(G)** Densitometric analysis of the WB experiments shown in **(F)**. The results are presented as the mean of *n* = 3 experiments, and the statistical analysis was performed using one-way ANOVA, followed by Dunn’s Multiple Comparison Test ^∗^*p* < 0.05.

The miRNAs recognition element for miR-101b in the 3′UTR of SynGAP (**Figure 1A**) corresponds to a canonical binding site (8-mer; **Figure 1B**) broadly conserved among mammals (**Figure 1C**). To validate the *in silico* prediction of SynGAP as a target of miR-101b, we introduced a miRNA mimic into the immortalized mouse hippocampal cell line, HT22, and evaluated the expression of endogenous SynGAP through WB. It is evident that the increase of miR-101b decreases the level of SynGAP in a dose-dependent manner (**Figures 1D,E**). This result validates the *in silico* prediction of SynGAP as a target of miR-101b and suggests that SynGAP expression could be modulated through Wnt-5a signaling via miR-101b. Considering that Wnt-5a signaling generates a significant decrease in the levels of miR-101b (Codocedo and Inestrosa, 2013), we reasoned that the levels of SynGAP could be increased in the presence of Wnt-5a. Using WB analyses, we determined that treatment with recombinant protein Wnt5a (rWnt-5a) increases the levels of SynGAP in a time-dependent manner in primary hippocampal neurons (**Figures 1F,G**) which could be due to the loss of translational repression exercised by miR-101b.

### Wnt-5a Rapidly Increases the Phosphorylation Levels of SynGAP, Modulating SynGAP Clusters

**FIGURE 2:**
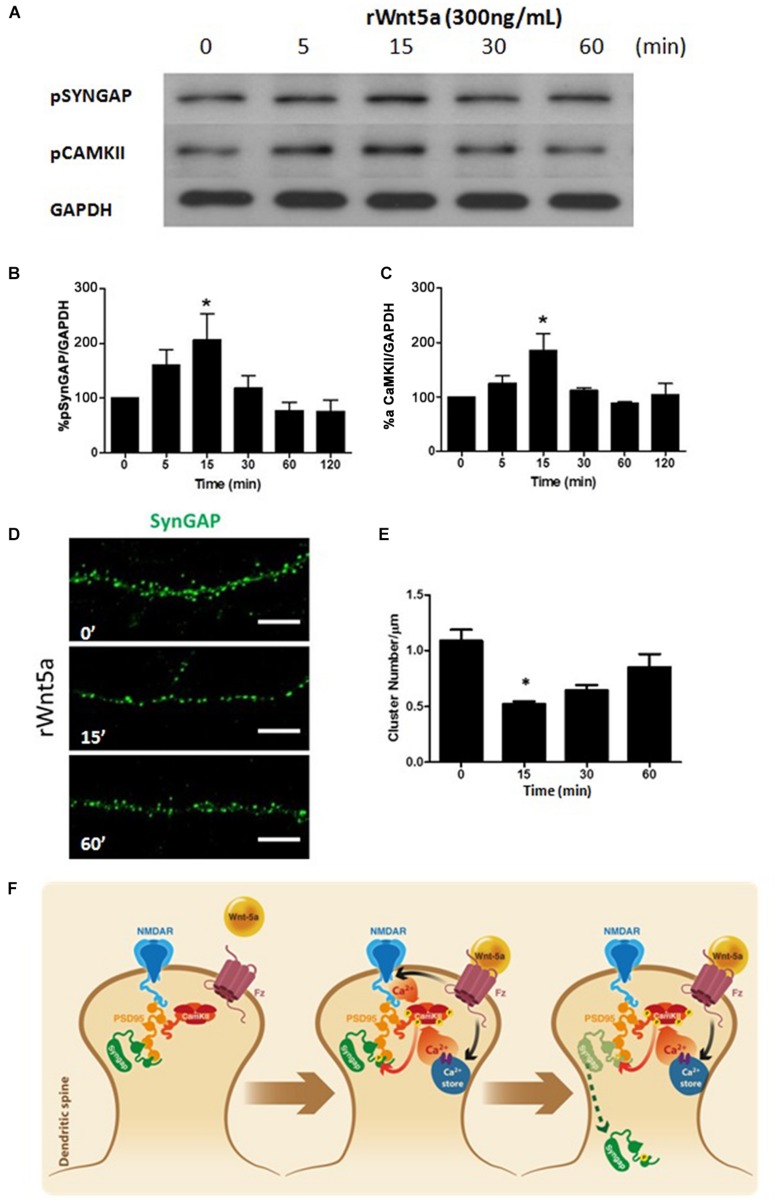
**Wnt-5a increases the phosphorylation levels of SynGAP by a mechanism dependent of CamKII. (A)** WB analysis of pSynGAP (S1123) and p-calmodulin-dependent protein kinase II (CaMKII) levels in hippocampal neurons treated with recombinant Wnt-5a (300 ng/mL) at different time points. **(B)** Densitometric analysis of pSynGAP1, shown in **(A)**. **(C)** Densitometric analysis of pCaMKII, shown in **(A)**. The results are presented as the mean of *n* = 3 experiments and were normalized to GAPDH expression. **(D)** Representative neurite images of SynGAP immunofluorescence (green) from samples subjected to rWnt-5a (300 ng/mL) treatment for different time points, white bar represents 5 μm. **(E)** Quantification of the cluster density of SynGAP (cluster number/μm) in neurons described in **(A)**. **(F)** Model of the effect of Wnt-5a on SynGAP function. After binding of Wnt-5a to their receptors, there is an increase of intracellular calcium which could be due to a release from internal stores (Wnt/Ca^+2^ pathway) or modulation of NMDAR. Activation of CaMKII induces the increase in phosphorylation levels of SynGAP at S1123 and migration from dendritic clusters to a diffuse pool in the dendritic shaft. Statistical analysis was performed using one-way ANOVA, followed by Dunn’s Multiple Comparison Test. ^∗^*p* < 0.05.

Synaptic GTPase-Activating Protein is a synaptic GTPase activating protein (GAP) that facilitates the hydrolysis of GTP to GDP and thereby negatively regulates the activity of RAS and RAP (Walkup et al., 2015). The GAP activity of SynGAP is increased by direct phosphorylation of serine residues by CaMKII (Oh et al., 2004), another prominent component of the PSD. Since Wnt-5a is able to increase calcium levels at dendritic compartments, activates CaMKII and has a significant role in the organization of the PSD (Farías et al., 2009). We evaluated whether Wnt-5a is able to activate SynGAP. The treatment with recombinant Wnt-5a generates a fast and transient increase in the phosphorylation levels of S1123 of SynGAP (**Figures 2A,B**), a major phosphorylation site for CaMKII (Oh et al., 2004). Additionally, the kinetics of this activation is similar to the activation of CaMKII after Wnt-5a treatment (**Figures 2A,C**).

Synaptic GTPase-Activating Protein is expressed only in neurons, including most excitatory neurons and a subset of inhibitory neurons (Zhang et al., 1999), where it is highly localized to the PSD (Chen et al., 1998). To evaluate the effects of Wnt-5a over the localization of SynGAP, we perform immunofluorescence studies on 14 DIV neurons treated with Wnt-5a at different time points.

Under control conditions, SynGAP showed a clear punctate localization (clusters) at dendrites which is consistent with previous studies on SynGAP’s enrichment in dendritic spines (Kim et al., 1998). Interestingly, upon Wnt-5a treatment, the amount of SynGAP clusters was significantly reduced (**Figures 2D,E**). This reduction occurred within 15–30 min after Wnt-5a treatment and was not fully recovered after 1 h of stimulation (**Figures 2D,E**). The reduction in SynGAP clusters, as shown above, was not due to decreased levels of total protein as demonstrated by WB analysis (**Figures 1F,G**). This suggests that the reduction in the SynGAP cluster number is due to a translocation from dendritic spines to a diffuse pool in the shaft of the dendrite.

As shown in the model (**Figure 2F**), SynGAP is tightly associated with the postsynaptic plasma membrane and binds to the PDZ domains of PSD-95 (Kim et al., 1998) which positions it in close proximity to the NMDA receptors. Previous reports show that SynGAP is rapidly dispersed from spines upon NMDAR activation (Araki et al., 2015). This dispersion is mediated by direct phosphorylation by CaMKII. In our hypothetical model, the activation of CaMKII mediated by Wnt-5a should be able to induce a reduction in the clusters of SynGAP in hippocampal neurons. This is probably the result of the translocation of SynGAP from relatively large clusters in spines, to a diffuse pool in the dendritic shaft.

### SynGAP Loss Induced by Aβ Oligomers is Prevented by Wnt-5a

**FIGURE 3:**
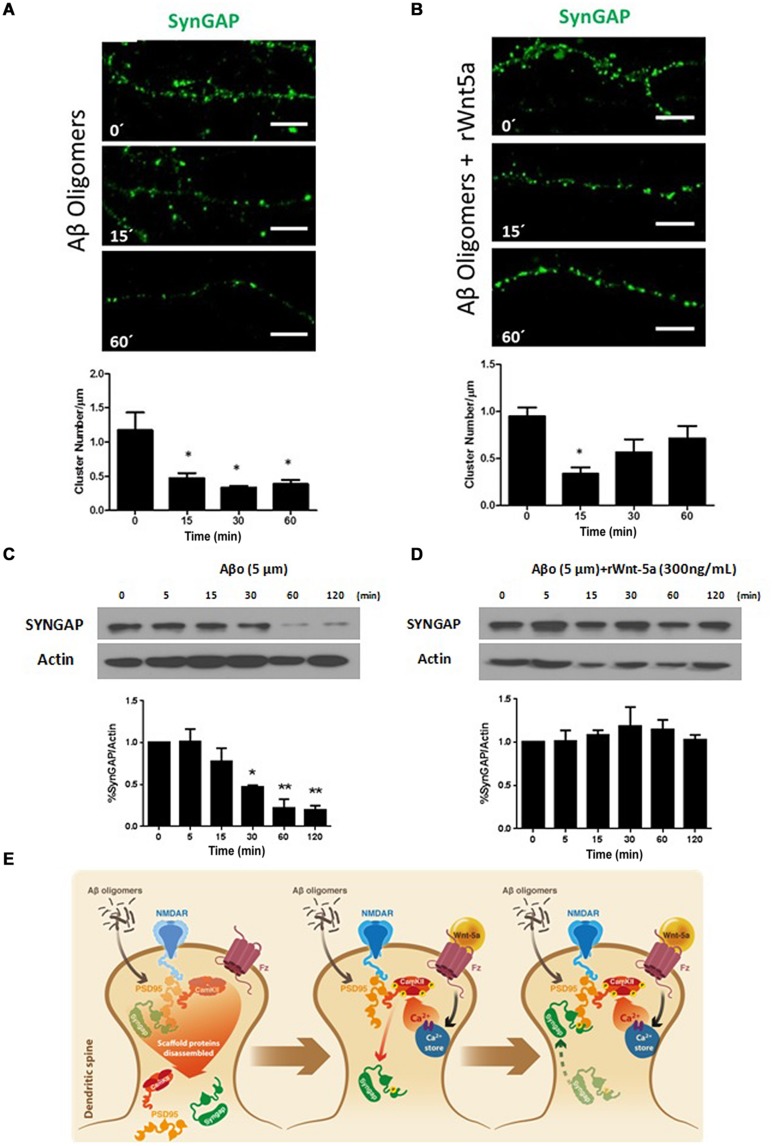
**SynGAP loss induced by Aβ oligomers is rescued by Wnt-5a. (A)** Representative neurite images of SynGAP immunofluorescence (green) from samples subjected to a Aβ oligomers (5 μM) treatment for different time points, white bar represents 5 μm. Below, quantification of SynGAP (cluster number/μm) after Aβ oligomers treatment, compared to control condition, as seen in **(A)**. Significant differences are found at 15, 30, and 60 min. **(B)** Representative neurite images of SynGAP immunofluorescence (green) from samples subjected a co-application of rWnt-5a (300 ng/mL) plus Aβ oligomers (5 μM) treatment for different time points, white bar represents 5 μm. Below, quantification of SynGAP (cluster number/μm) after Aβ oligomers plus rWnt-5a treatment, compared to control condition, as seen in **(B)**. Significant differences are found only at 15 min. **(C)** WB analysis of total SynGAP levels in hippocampal neurons treated with Aβ oligomers (5 μM) at different time points. **(D)** WB analysis of total SynGAP levels in hippocampal neurons treated with Aβ oligomers (5 μM) plus rWnt-5a (300 ng/mL) at different time points. **(E)** Model of the effect of Aβ oligomers and the protective effect of rWnt-5a. The results are presented as the mean of *n* = 3 and 4 experiments and the statistical analysis was performed using one-way ANOVA, followed by Dunn’s Multiple Comparison Test. ^∗^*p* < 0.05, ^∗∗^*p* < 0.005.

In the amyloid cascade hypothesis of AD, Aβ neurotoxicity has its origin in the binding of Aβ oligomers to the post-synaptic region (Hardy and Selkoe, 2002). Aβ directly affects synaptic components including PSD-95 (Roselli et al., 2005; Cerpa et al., 2010), NMDA receptors (Snyder et al., 2005), and α-amino-3-hydroxy-5-methyl-4-isoxazolepropionic acid receptors (AMPARs; Hsieh et al., 2006), to name a few (Dinamarca et al., 2012). Still, there is no evidence of an effect of Aβ oligomers on SynGAP function. To evaluate this possibility we treated neurons with 5 μM Aβ oligomers at different time points. At this concentration, Aβ oligomers do not induce significant neuronal cell death; in the time frame we performed the experiments, as revealed by Höechst staining (Supplementary Figure S1). This is in agreement with the idea that Aβ oligomers causes synaptic failure before neuronal death occurs (Hardy and Selkoe, 2002). Aβ oligomers treatment generates a fast and important decrease in SynGAP clusters per μm of dendrite, this effect seems to be permanent in time (**Figure 3A**). By WB analysis we evaluate the effect of Aβ oligomers on the SynGAP levels. The treatment with Aβ generates a significant decrease in the total amount of SynGAP (**Figure 3C**) which is consistent with the decrease in the dendritic clusters of SynGAP and the previously reported synaptic loss induced by Aβ oligomers (Dinamarca et al., 2008).

Previous studies indicate that Wnt-5a is able to prevent the Aβ synaptotoxicity triggered by the Aβ oligomers. Electrophysiological analysis of Schaffer collaterals-CA1 glutamatergic transmission in hippocampal slices demonstrated that Wnt-5a prevents the decrease in the amplitude of field excitatory postsynaptic potentials (_f_EPSPs). Moreover, Wnt-5a prevented the decrease of PSD-95 and synaptic loss in cultured hippocampal neurons (Cerpa et al., 2010). Additionally, the *in vivo* activation of Wnt signaling, with a mimetic peptide of Wnt-5a, rescues memory loss and improves synaptic dysfunction in APP/PS1-transgenic mice, a model the amyloid pathology of AD (Skaper, 2014; Vargas et al., 2014). Considering this evidence, we decided to evaluate the neuroprotective effects of Wnt-5a over SynGAP. The co-incubation of Wnt-5a with Aβ oligomers, reduced the number of apoptotic neurons at 120 min of treatment, time in which Aβ alone shows a slight increase in the apoptotic rate (Supplematary Figure S1). Hippocampal neurons treated with Aβ oligomers in the presence of the Wnt-5a ligand, showed a decrease in the SynGAP clusters (**Figure 3B**), very similar to the observed by the treatment with Wnt-5a alone (**Figures 2D,E**). At longer time of incubation, the SynGAP cluster number shows an increase close to control levels (**Figure 3B**), which is not observed in neurons treated with Aβ oligomers alone (**Figure 3A**). Interestingly, by WB analysis we observe that the total level of SynGAP is not altered when neurons were treated with Aβ oligomers in the presence of Wnt-5a (**Figure 3D**). This contrast with the effects observed in the cluster number of SynGAP when Wnt-5a is co-incubated with Aβ oligomers, in which we observe an inicial decrease. This suggest that the decrease observed at 5 min could be product of a translocation of SynGAP and not a loss in the protein level.

As shown in the model (**Figure 3E**), our results suggests that Aβ oligomers cause a disarrangement of SynGAP organization, which could be due to the PSD disassembling (Dinamarca et al., 2008, 2012). In our hypothetical model, Wnt-5a prevents the SynGAP loss induced by Aβ oligomers treatment by maintaining the integrity of the PSD and dendritic spines as previously has been reported (Cerpa et al., 2010; Varela-Nallar et al., 2012). This allow that the initial decrease observed for SynGAP cluster density in the presence of Wnt-5a and Aβ oligomers (15 min) return to control conditions at longer times of incubations.

## Discussion

Wnt-5a is a synaptogenic factor, whose expression increases during development. It has been suggested that Wnt-5a plays a key role in synaptic function in the adult nervous system (Inestrosa and Arenas, 2010; Inestrosa and Varela-Nallar, 2014). Emerging *in vitro* and *in vivo* studies have implicated Wnt signaling in synaptic plasticity, regulating LTP (Cerpa et al., 2011), and episodic memory (Vargas et al., 2014). Furthermore, activation of Wnt-5a signaling has shown to protect against Aβ-induced synaptic impairment (Cerpa et al., 2010; Varela-Nallar et al., 2012; Vargas et al., 2014). However, the molecular targets of their activity are just beginning to be elucidated.

Synaptic GTPase-Activating Protein is a prominent Ras/Rap GTPase-activating protein located in the PSD. It regulates the timing of spine formation and trafficking of glutamate receptors in cultured neurons (Rumbaugh et al., 2006). The role of SynGAP in plastic processes has been well established. SYNGAP1 knockout mice show deficits in NMDAR-dependent LTP (Kim et al., 2003) and have deficits in learning and memory (Clement et al., 2012).

In the present study, we evaluated the hypothesis that treatment with Wnt-5a modulates SynGAP function in hippocampal neurons, considering first, that SynGAP is a predicted target of a Wnt-5a-regulated microRNA, miR-101b, and second, that CaMKII activity is a major component in the signaling of both proteins. Our results show that endogenous SynGAP is down-regulated in HT-22 cells transfected with miR-101b mimic which validate the *in silico* prediction. In agreement with this observation and considering that Wnt-5a generates a significant decrease in the levels of miR-101b (Codocedo and Inestrosa, 2013), the treatment with Wnt-5a generates an increases in the total levels of SynGAP in hippocampal neurons at 1 h of stimulation. The meaning of this regulation could be related to the increase in protein translation observed in synaptic plasticity process (Kelleher et al., 2004; Schuman et al., 2006; Costa-Mattioli et al., 2009), however, additional experiments are required to confirm this hypothesis.

The GAP activity of SynGAP is increased by direct phosphorylation of serine residues by CaMKII (Oh et al., 2004). The treatment with Wnt-5a, increases the phosphorylation of S1123 residue of SynGAP, a major phosphorylation target of CaMKII. Additionally, we observed that the kinetics of this effect is similar to the activation of CaMKII induced by Wnt-5a. Both proteins increased their levels with a peak in 15 min and then return to basal levels. This finding is interesting because it suggests that the increase in intracellular calcium levels mediated by Wnt-5a might activate SynGAP. In the literature, is normally accepted that the calcium source that results in SynGAP activation is the influx mediated by NMDAR, posterior to LTP induction (Krapivinsky et al., 2004; Rumbaugh et al., 2006; Araki et al., 2015). In the Wnt/Ca^2+^ pathway, the Wnt-5a ligand activates PKC, CaMKII and calcineurin, by increasing the intracellular calcium concentration coming from internal stores (Kühl, 2004; Kohn and Moon, 2005; Muñoz et al., 2014), suggesting a mechanism independent of NMDAR activation. However, is possible that the Wnt/Ca^2+^ pathway activate SynGAP through NMDAR modulation. Recent reports show that Wnt-5a increases the GluN2B subunit of the NMDAR on the hippocampal neuronal cell surface (Muñoz et al., 2014). Additionally, Wnt-5a acutely and specifically upregulates synaptic NMDAR currents in rat hippocampal slices (Cerpa et al., 2011). Further studies are necessary to determine if the mechanism of activation of SynGAP mediated by Wnt-5a, is independent of NMDAR activation or could result from NMDAR modulation.

The functional consequence of SynGAP activation has been highly debated because RAS and RAP, their major targets at the synapse, have opposite effects in synaptic strength modulation. Active Ras increases insertion (exocytosis) of AMPARs at the synapse; whereas, active Rap increases their removal (endocytosis) from the synapse (Zhu et al., 2002). Additionally, SynGAP has also been reported to have a promiscuous GTPase activity that directly or indirectly regulates the small G-proteins Rac1, and Rab5, which may in turn regulate the actin cytoskeleton and membrane trafficking involved in LTP-induced increases in spine size and AMPAR recruitment (Carlisle et al., 2008). Wnt-5a signaling induces a strengthening of synapses which is inconsistent with the Ras inhibition induced by SynGAP. However, a recent report shows that differential phosphorylation of SynGAP by CaMKII and CDK5 may alter the proportions of activated Ras and Rap in synapses with consequent effects on the cellular processes regulated by the two GTPases (Walkup et al., 2015). Phosphorylation of SynGAP by CaMKII at S1123 accelerates the rate of inactivation of Rap more potently than the rate of inactivation of Ras; whereas, phosphorylation by CDK5, which occurs at S773 and S802, has the opposite effect (Walkup et al., 2015). Another interesting mechanism of regulation of SynGAP mediated by CaMKII was recently described, in which their phosphorylation triggers a rapid dispersion of SynGAP clusters from synaptic spines, activating synaptic Ras and inducing LTP (Araki et al., 2015). We observe a fast and significative reduction of SynGAP cluster number after Wnt-5a treatment, without a reduction in SynGAP protein levels, which is consistent with a translocation of SynGAP from aggregates (presumably dendritic spines) to a diffuse pool in dendritic shaft. The timing of reduction in cluster number is similar to the peak of phosphorylation of SynGAP and CaMKII. The cluster number returns to basal levels more slowly than the dephosphorylation of SynGAP and CaMKII. This suggests that this post-transcriptional modification is necessary for cluster translocation. However, the re-insertion of the clusters in the PSD could implicate additional mechanisms.

In addition to its synaptic role, Wnt-5a is able to protect neurons against Aβ oligomers synaptotoxicity. Deregulation of the Wnt signaling has been suggested as an etiological cause for AD (Inestrosa and Arenas, 2010), which correspond to the most common type of dementia in people over 65 years old, in which death or malfunction of neurons causes changes in memory, behavior, and cognition (Thies and Bleiler, 2013). Synaptic pathology is an early event in AD, and soluble Aβ oligomers are principal responsible for the synaptic failure. Long before the occurrence of plaque deposition and neuronal death (Walsh and Selkoe, 2007). Activation of Wnt-5a signaling rescues memory loss and improves synaptic dysfunction in both *in vivo* and *in vitro* models of AD (Cerpa et al., 2010; Varela-Nallar et al., 2012; Skaper, 2014; Vargas et al., 2014). Considering the importance of SynGAP signaling in plastic and cognitive processes, it results surprising the absence of reports studying the role of SynGAP in AD mice models. For this reason, we evaluate the clustering of SynGAP in the presence of Aβ oligomers. The treatment with 5 μM of Aβ oligomers generates a fast and significant decrease in SynGAP level which becomes more pronounced in time. The initial decrease in SynGAP cluster number could be attributable to an increase in intracellular calcium levels, as has been reported previously for Aβ oligomers (Kawahara, 2010). However, the fact that the cluster number continues to decrease, as opposed to what is observed with Wnt-5a, suggests that the reduction is the result of synaptic toxicity. Interestingly, the co-application of Wnt-5a prevents this toxic effect. This probably explains why, when Aβ oligomers are co-applied with Wnt-5a we do not observe a decrease in SynGAP clusters. Moreover, it shows a similar effect to the one observed for Wnt-5a treatments. This observation is also similar to the previously reported effects of Wnt-5a in the clustering of PSD-95. Which drastically decreased in the presence of Aβ oligomers, while the co-incubation with Wnt-5a prevented such changes (Cerpa et al., 2010). The loss of SynGAP clusters in the presence of Aβ oligomers, could be due to the loss of PSD-95, considering that this scaffolding protein binds SynGAP through their PDZ (Postsynaptic Density Protein-95, *Drosophila* Disk Large Tumor Suppressor, and Zonula Occludens-1) domains. The fact that Wnt-5a prevents the loss of PSD-95 and dendritic spine structure in the presence of Aβ oligomers, helps to preserve the platform that allow the recovery of SynGAP clustering. Additional experiments are necessary to evaluate further mechanisms involved in the regulation of this phenomenon.

In the present work, we show for the first time the effect of Wnt-5a ligand over SynGAP function. Wnt-5a regulates the expression of SynGAP by a mechanism that could involve miRNA modulation. Additionally Wnt-5a regulates the phosphorylation levels (activity) and localization of SynGAP. Finally, we report that Aβ oligomers disturb the distribution of SynGAP and that Wnt-5a can protects against this synaptotoxic effect.

## Conflict of Interest Statement

The authors declare that the research was conducted in the absence of any commercial or financial relationships that could be construed as a potential conflict of interest.

## References

[B1] Alvarez A. R. Godoy J. A. Mullendorff K. Olivares G. H. Bronfman M. Inestrosa N. C. (2004). Wnt-3a overcomes beta-amyloid toxicity in rat hippocampal neurons. *Exp. Cell Res.* 297 186–196. 10.1016/j.yexcr.2004.02.028 15194435

[B2] Araki Y. Zeng M. Zhang M. Huganir R. L. (2015). Rapid dispersion of SynGAP from synaptic spines triggers AMPA receptor insertion and spine enlargement during LTP. *Neuron* 85 173–189. 10.1016/j.neuron.2014.12.023 25569349 PMC4428669

[B3] Barbato C. Pezzola S. Caggiano C. Antonelli M. Frisone P. Ciotti M. T. (2014). A lentiviral sponge for miR-101 regulates RanBP9 expression and amyloid precursor protein metabolism in hippocampal neurons. *Front. Cell. Neurosci.* 8:37 10.3389/fncel.2014.00037 PMC3923151 24592211

[B4] Bartel D. P. (2009). MicroRNAs: target recognition and regulatory functions. *Cell* 136 215–233. 10.1016/j.cell.2009.01.002 19167326 PMC3794896

[B5] Berryer M. H. Hamdan F. F. Klitten L. L. Møller R. S. Carmant L. Schwartzentruber J. (2013). Mutations in SYNGAP1 cause intellectual disability, autism, and a specific form of epilepsy by inducing haploinsufficiency. *Hum. Mutat.* 34 385–394. 10.1002/humu.22248 23161826

[B6] Budnik V. Salinas P. C. (2011). Wnt signaling during synaptic development and plasticity. *Curr. Opin. Neurobiol.* 21 151–159. 10.1016/j.conb.2010.12.002 21239163 PMC3499977

[B7] Carlisle H. J. Manzerra P. Marcora E. Kennedy M. B. (2008). SynGAP regulates steady-state and activity-dependent phosphorylation of cofilin. *J. Neurosci.* 28 13673–13683. 10.1523/JNEUROSCI.4695-08.2008 19074040 PMC2615239

[B8] Cerpa W. Farías G. G. Godoy J. A. Fuenzalida M. Bonansco C. Inestrosa N. C. (2010). Wnt-5a occludes Abeta oligomer-induced depression of glutamatergic transmission in hippocampal neurons. *Mol. Neurodegener.* 5:3 10.1186/1750-1326-5-3 PMC2823745 20205789

[B9] Cerpa W. Gambrill A. Inestrosa N. C. Barria A. (2011). Regulation of NMDA-receptor synaptic transmission by Wnt signaling. *J. Neurosci.* 31 9466–9471. 10.1523/JNEUROSCI.6311-10.2011 21715611 PMC3141819

[B10] Chen H. J. Rojas-Soto M. Oguni A. Kennedy M. B. (1998). A synaptic Ras-GTPase activating protein (p135 SynGAP) inhibited by CaM kinase II. *Neuron* 20 895–904. 10.1016/S0896-6273(00)80471-7 9620694

[B11] Chen Y. Sabatini B. L. (2012). Signaling in dendritic spines and spine microdomains. *Curr. Opin. Neurobiol.* 22 389–396. 10.1016/j.conb.2012.03.003 22459689 PMC3391315

[B12] Chhunchha B. Fatma N. Kubo E. Rai P. Singh S. P. Singh D. P. (2013). Curcumin abates hypoxia-induced oxidative stress based-ER stress-mediated cell death in mouse hippocampal cells (HT22) by controlling Prdx6 and NF-κB regulation. *Am. J. Physiol. Cell Physiol.* 304 C636–C655. 10.1152/ajpcell.00345.2012 23364261 PMC3625714

[B13] Ciani L. Boyle K. A. Dickins E. Sahores M. Anane D. Lopes D. M. (2011). Wnt7a signaling promotes dendritic spine growth and synaptic strength through Ca^2+^/Calmodulin-dependent protein kinase II. *Proc. Natl. Acad. Sci. U.S.A.* 108 10732–10737. 10.1073/pnas.1018132108 21670302 PMC3127879

[B14] Clement J. P. Aceti M. Creson T. K. Ozkan E. D. Shi Y. Reish N. J. (2012). Pathogenic SYNGAP1 mutations impair cognitive development by disrupting maturation of dendritic spine synapses. *Cell* 151 709–723. 10.1016/j.cell.2012.08.045 23141534 PMC3500766

[B15] Codocedo J. F. Allard C. Godoy J. A. Varela-Nallar L. Inestrosa N. C. (2012). SIRT1 regulates dendritic development in hippocampal neurons. *PLoS ONE* 7:e47073 10.1371/journal.pone.0047073 PMC3464248 23056585

[B16] Codocedo J. F. Inestrosa N. C. (2013). “Wnt5a regulates expression of ROCK2 and SYNGAP1 through Mir-101b in rat hippocampal neurons,” in *Proceedings of the Program No. 223.16/C11. 2013 Neuroscience Meeting Planner. Society for Neuroscience, Nov 9-13, 2013* San Diego, CA.

[B17] Colgan L. A. Yasuda R. (2014). Plasticity of dendritic spines: subcompartmentalization of signaling. *Annu. Rev. Physiol.* 76 365–385. 10.1146/annurev-physiol-021113-170400 24215443 PMC4142713

[B18] Costa-Mattioli M. Sossin W. S. Klann E. Sonenberg N. (2009). Translational control of long-lasting synaptic plasticity and memory. *Neuron* 61 10–26. 10.1016/j.neuron.2008.10.055 19146809 PMC5154738

[B19] Cuitino L. Godoy J. A. Farías G. G. Couve A. Bonansco C. Fuenzalida M. (2010). Wnt-5a modulates recycling of functional GABAA receptors on hippocampal neurons. *J. Neurosci.* 30 8411–8420. 10.1523/JNEUROSCI.5736-09.2010 20573888 PMC6634644

[B20] Dinamarca M. C. Colombres M. Cerpa W. Bonansco C. Inestrosa N. C. (2008). Beta-amyloid oligomers affect the structure and function of the postsynaptic region: role of the Wnt signaling pathway. *Neurodegener. Dis.* 5 149–152. 10.1159/000113687 18322375

[B21] Dinamarca M. C. Ríos J. A. Inestrosa N. C. (2012). Postsynaptic receptors for amyloid-β oligomers as mediators of neuronal damage in Alzheimer’s disease. *Front. Physiol.* 3:464 10.3389/fphys.2012.00464 PMC3526732 23267328

[B22] Farías G. G. Alfaro I. E. Cerpa W. Grabowski C. P. Godoy J. A. Bonansco C. (2009). Wnt-5a/JNK signaling promotes the clustering of PSD-95 in hippocampal neurons. *J. Biol. Chem.* 284 15857–15866. 10.1074/jbc.M808986200 19332546 PMC2708882

[B23] Gogolla N. Galimberti I. Deguchi Y. Caroni P. (2009). Wnt signaling mediates experience-related regulation of synapse numbers and mossy fiber connectivities in the adult hippocampus. *Neuron* 62 510–525. 10.1016/j.neuron.2009.04.022 19477153

[B24] Hamzeiy H. Allmer J. Yousef M. (2014). Computational Methods for MicroRNA Target Prediction. *Methods Mol. Biol.* 1107 207–221. 10.1007/978-1-62703-748-8_12 24272439

[B25] Hao Y. Gu X. Zhao Y. Greene S. Sha W. Smoot D. T. (2011). Enforced expression of miR-101 inhibits prostate cancer cell growth by modulating the COX-2 pathway in vivo. *Cancer Prev. Res. (Phila).* 4 1073–1083. 10.1158/1940-6207.CAPR-10-0333 21430074 PMC3305792

[B26] Hardy J. Selkoe D. J. (2002). The amyloid hypothesis of Alzheimer’s disease: progress and problems on the road to therapeutics. *Science* 297 353–356. 10.1126/science.1072994 12130773

[B27] He X.-P. Shao Y. Li X.-L. Xu W. Chen G.-S. Sun H.-H. (2012). Downregulation of miR-101 in gastric cancer correlates with cyclooxygenase-2 overexpression and tumor growth. *FEBS J.* 279 4201–4212. 10.1111/febs.12013 23013439

[B28] Hsieh H. Boehm J. Sato C. Iwatsubo T. Tomita T. Sisodia S. (2006). AMPAR removal underlies ABeta-induced synaptic depression and dendritic spine loss. *Neuron* 52 831–843. 10.1016/j.neuron.2006.10.035 17145504 PMC1850952

[B29] Inestrosa N. C. Arenas E. (2010). Emerging roles of Wnts in the adult nervous system. *Nat. Rev. Neurosci.* 11 77–86. 10.1038/nrn2755 20010950

[B30] Inestrosa N. C. Montecinos-Oliva C. Fuenzalida M. (2012). Wnt signaling: role in Alzheimer disease and schizophrenia. *J. Neuroimmune Pharmacol.* 7 788–807. 10.1007/s11481-012-9417-5 23160851

[B31] Inestrosa N. C. Toledo E. M. (2008). The role of Wnt signaling in neuronal dysfunction in Alzheimer’s Disease. *Mol. Neurodegener.* 3:9 10.1186/1750-1326-3-9 PMC2515306 18652670

[B32] Inestrosa N. C. Varela-Nallar L. (2014). Wnt signaling in the nervous system and in Alzheimer’s disease. *J. Mol. Cell Biol.* 6 64–74. 10.1093/jmcb/mjt051 24549157

[B33] Kaech S. Banker G. (2006). Culturing hippocampal neurons. *Nat. Protoc.* 1 2406–2415. 10.1038/nprot.2006.356 17406484

[B34] Kawahara M. (2010). Neurotoxicity of β-amyloid protein: oligomerization, channel formation, and calcium dyshomeostasis. *Curr. Pharm. Des.* 16 2779–2789. 10.2174/138161210793176545 20698821

[B35] Kelleher R. J. Govindarajan A. Tonegawa S. (2004). Translational regulatory mechanisms in persistent forms of synaptic plasticity. *Neuron* 44 59–73. 10.1016/j.neuron.2004.09.013 15450160

[B36] Kim J. H. Lee H.-K. Takamiya K. Huganir R. L. (2003). The role of synaptic GTPase-activating protein in neuronal development and synaptic plasticity. *J. Neurosci.* 23 1119–1124. 12598599 10.1523/JNEUROSCI.23-04-01119.2003 PMC6742247

[B37] Kim J. H. Liao D. Lau L. F. Huganir R. L. (1998). SynGAP: A synaptic RasGAP that associates with the PSD-95/SAP90 protein family. *Neuron* 20 683–691. 10.1016/S0896-6273(00)81008-9 9581761

[B38] Kohn A. D. Moon R. T. (2005). Wnt and calcium signaling: beta-catenin-independent pathways. *Cell Calcium* 38 439–446. 10.1016/j.ceca.2005.06.022 16099039

[B39] Krapivinsky G. Medina I. Krapivinsky L. Gapon S. Clapham D. E. (2004). SynGAP-MUPP1-CaMKII synaptic complexes regulate p38 MAP kinase activity and NMDA receptor-dependent synaptic AMPA receptor potentiation. *Neuron* 43 563–574. 10.1016/j.neuron.2004.08.003 15312654

[B40] Kühl M. (2004). The WNT/calcium pathway: biochemical mediators, tools and future requirements. *Front. Biosci.* 9:967–974. 10.2741/1307 14766423

[B41] Lee Y. Samaco R. C. Gatchel J. R. Thaller C. Orr H. T. Zoghbi H. Y. (2008). miR-19, miR-101 and miR-130 co-regulate ATXN1 levels to potentially modulate SCA1 pathogenesis. *Nat. Neurosci.* 11 1137–1139. 10.1038/nn.2183 18758459 PMC2574629

[B42] Lewis B. P. Burge C. B. Bartel D. P. (2005). Conserved seed pairing, often flanked by adenosines, indicates that thousands of human genes are microRNA targets. *Cell* 120 15–20. 10.1016/j.cell.2004.12.035 15652477

[B43] Lie D.-C. Colamarino S. A. Song H.-J. Désiré L. Mira H. Consiglio A. (2005). Wnt signalling regulates adult hippocampal neurogenesis. *Nature* 437 1370–1375. 10.1038/nature04108 16251967

[B44] Long J. M. Lahiri D. K. (2011). MicroRNA-101 downregulates Alzheimer’s amyloid-β precursor protein levels in human cell cultures and is differentially expressed. *Biochem. Biophys. Res. Commun*. 404 889–895. 10.1016/j.bbrc.2010.12.053 21172309 PMC3372402

[B45] Lovestone S. Killick R. Di Forti M. Murray R. (2007). Schizophrenia as a GSK-3 dysregulation disorder. *Trends Neurosci.* 30 142–149. 10.1016/j.tins.2007.02.002 17324475

[B46] McMahon A. C. Barnett M. W. O’Leary T. S. Stoney P. N. Collins M. O. Papadia S. (2012). SynGAP isoforms exert opposing effects on synaptic strength. *Nat. Commun.* 3:900 10.1038/ncomms1900 PMC3621422 22692543

[B47] Muñoz F. J. Godoy J. A. Cerpa W. Poblete I. M. Huidobro-Toro J. P. Inestrosa N. C. (2014). Wnt-5a increases NO and modulates NMDA receptor in rat hippocampal neurons. *Biochem. Biophys. Res. Commun.* 444 189–194. 10.1016/j.bbrc.2014.01.031 24440698

[B48] Nicoloso M. S. Spizzo R. Shimizu M. Rossi S. Calin G. A. (2009). MicroRNAs–the micro steering wheel of tumour metastases. *Nat. Rev. Cancer* 9 293–302. 10.1038/nrc2619 19262572

[B49] Nusse R. Varmus H. (2012). Three decades of Wnts: a personal perspective on how a scientific field developed. *EMBO J*. 31 2670–2684. 10.1038/emboj.2012.146 22617420 PMC3380217

[B50] Oh J. S. Manzerra P. Kennedy M. B. (2004). Regulation of the Neuron-specific Ras GTPase-activating Protein, synGAP, by Ca^2+^/Calmodulin-dependent Protein Kinase II. *J. Biol. Chem.* 279 17980–17988. 10.1074/jbc.M314109200 14970204

[B51] Purro S. A. Dickins E. M. Salinas P. C. (2012). The secreted Wnt antagonist Dickkopf-1 is required for amyloid β-mediated synaptic loss. *J. Neurosci.* 32 3492–3498. 10.1523/JNEUROSCI.4562-11.2012 22399772 PMC6621056

[B52] Roselli F. Tirard M. Lu J. Hutzler P. Lamberti P. Livrea P. (2005). Soluble beta-amyloid1-40 induces NMDA-dependent degradation of postsynaptic density-95 at glutamatergic synapses. *J. Neurosci.* 25 11061–11070. 10.1523/JNEUROSCI.3034-05.2005 16319306 PMC6725651

[B53] Rumbaugh G. Adams J. P. Kim J. H. Huganir R. L. (2006). SynGAP regulates synaptic strength and mitogen-activated protein kinases in cultured neurons. *Proc. Natl. Acad. Sci. U.S.A.* 103 4344–4351. 10.1073/pnas.0600084103 16537406 PMC1450173

[B54] Schuman E. M. Dynes J. L. Steward O. (2006). Synaptic regulation of translation of dendritic mRNAs. *J. Neurosci.* 26 7143–7146. 10.1523/JNEUROSCI.1796-06.2006 16822969 PMC6673937

[B55] Silva-Alvarez C. Arrázola M. S. Godoy J. A. Ordenes D. Inestrosa N. C. (2013). Canonical Wnt signaling protects hippocampal neurons from Aβ oligomers: role of non-canonical Wnt-5a/Ca(2+) in mitochondrial dynamics. *Front. Cell. Neurosci.* 7:97 10.3389/fncel.2013.00097 PMC3691552 23805073

[B56] Skaper S. D. (2014). Wnt-signalling: a new direction for alzheimer disease? *CNS Neurol. Disord. Drug Targets* 13:556 10.2174/187152731304140702104826 25133285

[B57] Snyder E. M. Nong Y. Almeida C. G. Paul S. Moran T. Choi E. Y. (2005). Regulation of NMDA receptor trafficking by amyloid-beta. *Nat. Neurosci.* 8 1051–1058. 10.1038/nn1503 16025111

[B58] Sowers L. P. Loo L. Wu Y. Campbell E. Ulrich J. D. Wu S. (2013). Disruption of the non-canonical Wnt gene PRICKLE2 leads to autism-like behaviors with evidence for hippocampal synaptic dysfunction. *Mol. Psychiatry* 18 1077–1089. 10.1038/mp.2013.71 23711981 PMC4163749

[B59] Strillacci A. Griffoni C. Sansone P. Paterini P. Piazzi G. Lazzarini G. (2009). MiR-101 downregulation is involved in cyclooxygenase-2 overexpression in human colon cancer cells. *Exp. Cell Res.* 315 1439–1447. 10.1016/j.yexcr.2008.12.010 19133256

[B60] Thies W. Bleiler L. (2013). 2013 Alzheimer’s disease facts and figures. *Alzheimers Dement* 9 208–245. 10.1016/j.jalz.2013.02.003 23507120

[B61] Varela-Nallar L. Alfaro I. E. Serrano F. G. Parodi J. Inestrosa N. C. (2010). Wingless-type family member 5A (Wnt-5a) stimulates synaptic differentiation and function of glutamatergic synapses. *Proc. Natl. Acad. Sci. U.S.A.* 107 21164–21169. 10.1073/pnas.1010011107 21084636 PMC3000271

[B62] Varela-Nallar L. Grabowski C. P. Alfaro I. E. Alvarez A. R. Inestrosa N. C. (2009). Role of the Wnt receptor Frizzled-1 in presynaptic differentiation and function. *Neural Dev.* 4:41 10.1186/1749-8104-4-41 PMC2779803 19883499

[B63] Varela-Nallar L. Inestrosa N. C. (2013). Wnt signaling in the regulation of adult hippocampal neurogenesis. *Front. Cell. Neurosci.* 7:100 10.3389/fncel.2013.00100 PMC3693081 23805076

[B64] Varela-Nallar L. Parodi J. Farías G. G. Inestrosa N. C. (2012). Wnt-5a is a synaptogenic factor with neuroprotective properties against Aβ toxicity. *Neurodegener. Dis.* 10 23–26. 10.1159/000333360 22261402

[B65] Vargas J. Y. Fuenzalida M. Inestrosa N. C. (2014). In vivo activation of Wnt signaling pathway enhances cognitive function of adult mice and reverses cognitive deficits in an Alzheimer’s disease model. *J. Neurosci*. 34 2191–2202. 10.1523/JNEUROSCI.0862-13.2014 24501359 PMC6608527

[B66] Vilardo E. Barbato C. Ciotti M. Cogoni C. Ruberti F. (2010). MicroRNA-101 regulates amyloid precursor protein expression in hippocampal neurons. *J. Biol. Chem.* 285 18344–18351. 10.1074/jbc.M110.112664 20395292 PMC2881760

[B67] Walkup W. G. Washburn L. R. Sweredoski M. J. Carlisle H. J. Graham R. L. Hess S. (2015). Phosphorylation of synaptic GTPase activating protein (synGAP) by Ca^2+^/calmodulin-dependent protein kinase II (CaMKII) and cyclin-dependent kinase 5 (CDK5) alters the ratio of its GAP activity toward Ras and Rap GTPases. *J. Biol. Chem.* 290 4908–4927. 10.1074/jbc.M114.614420 25533468 PMC4335230

[B68] Walsh D. M. Selkoe D. J. (2007). A beta oligomers - a decade of discovery. *J. Neurochem.* 101 1172–1184. 10.1111/j.1471-4159.2006.04426.x 17286590

[B69] Zhang W. Vazquez L. Apperson M. Kennedy M. B. (1999). Citron binds to PSD-95 at glutamatergic synapses on inhibitory neurons in the hippocampus. *J. Neurosci.* 19 96–108. 9870942 10.1523/JNEUROSCI.19-01-00096.1999 PMC6782379

[B70] Zhang Y. Sun Y. Wang F. Wang Z. Peng Y. Li R. (2012). Downregulating the canonical Wnt/β-catenin signaling pathway attenuates the susceptibility to autism-like phenotypes by decreasing oxidative stress. *Neurochem. Res.* 37 1409–1419. 10.1007/s11064-012-0724-2 22374471

[B71] Zhu J. J. Qin Y. Zhao M. Van Aelst L. Malinow R. (2002). Ras and Rap control AMPA receptor trafficking during synaptic plasticity. *Cell* 110 443–455. 10.1016/S0092-8674(02)00897-8 12202034

[B72] Zongaro S. Hukema R. D’Antoni S. Davidovic L. Barbry P. Catania M. V. (2013). The 3’ UTR of FMR1 mRNA is a target of miR-101, miR-129-5p and miR-221: implications for the molecular pathology of FXTAS at the synapse. *Hum. Mol. Genet.* 22 1971–1982. 10.1093/hmg/ddt044 23390134

