# Supplementary figures and images for: Wnt-related SynGAP1 is a neuroprotective factor of glutamatergic synapses against Aβ oligomers
Source: Front Cell Neurosci. 2015 Jun 15;9:227. doi: 10.3389/fncel.2015.00227 (PMC4466443; doi:10.3389/fncel.2015.00227)

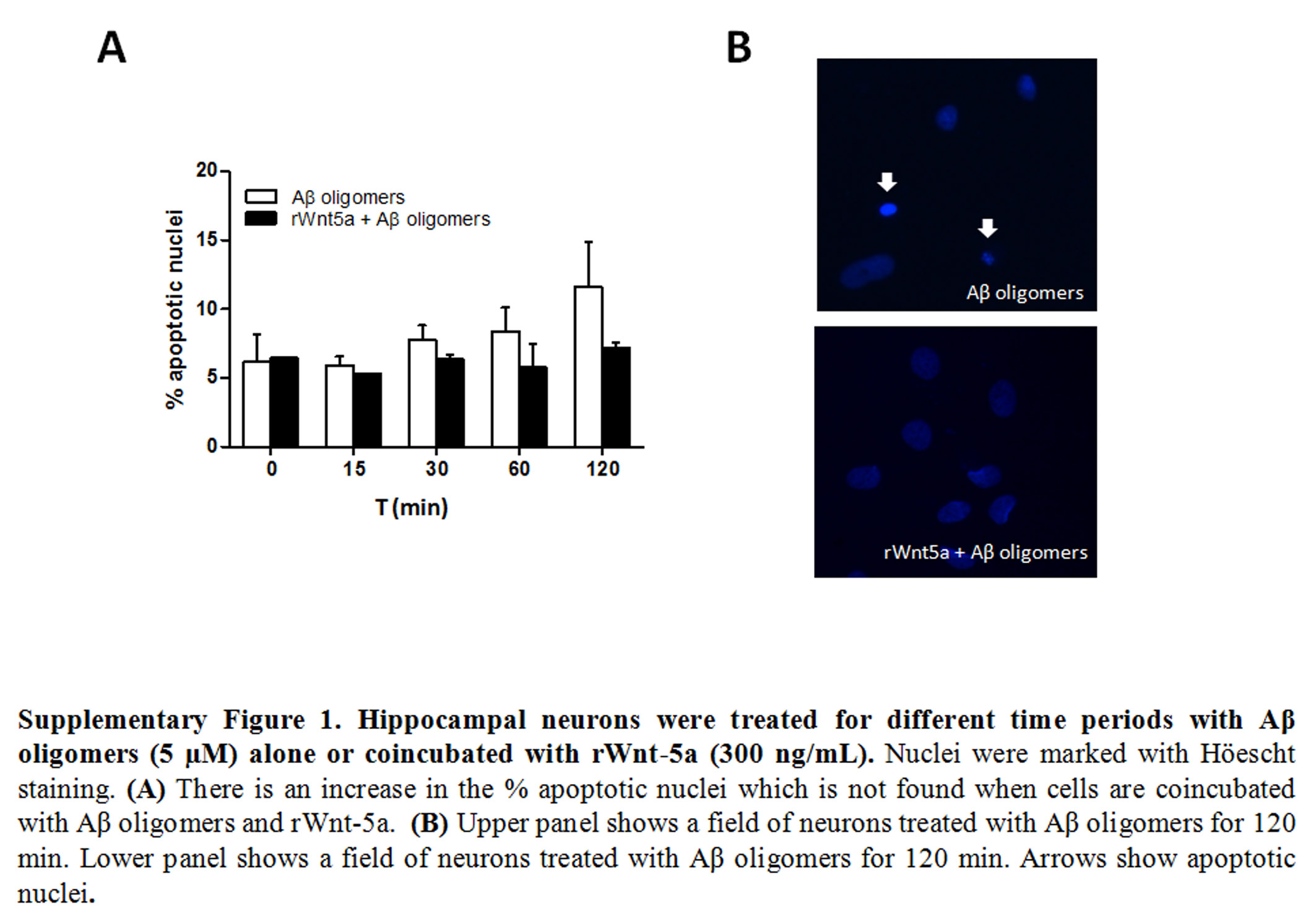

Supplement: Supplementary file 1 [file Image_1.JPEG]
